# EV-idence uncovered: kidney-on-chip study links circulating EVs of cardiorenal syndrome to renal pathology

**DOI:** 10.20517/evcna.2024.70

**Published:** 2024-10-26

**Authors:** Hawa Ndiaye, Simran Rajput, John F.K. Sauld, Gautam Mahajan, Saumya Das, Emeli Chatterjee

**Affiliations:** ^1^Cardiovascular Research Center, Massachusetts General Hospital, Boston, MA 02114, USA.; ^2^Department of Biology, College of Arts and Sciences, Boston University, Boston, MA 02215, USA.; ^3^Department of Microbiology, San Juan Bautista School of Medicine, Caguas, PR 00727, USA.; ^4^Emulate, Inc, Boston, MA 02210, USA.

**Keywords:** Heart failure, cardiorenal syndrome, extracellular vesicles, kidney-on-Chip, miRNA

## Abstract

The intertwined nature of cardiac and renal failure, where dysfunction in one organ predicts a poor outcome in the other, has long driven the interest in uncovering the exact molecular links between the two. Elucidating the mechanisms driving Cardiorenal Syndrome (CRS) will enable the development of targeted therapies that disrupt this detrimental cycle, potentially improving outcomes for patients. A recent study by Chatterjee *et al*. (JCI insight 2023) demonstrated the feasibility of utilizing a humanized microfluidic kidney-on-chip model to elucidate the role of circulating extracellular vesicles (EVs) in the development of CRS (type 1 and type 2) in heart failure (HF) patients. The study also identified and validated EV miRNAs that correlated with kidney function by targeting several genes involved in kidney damage pathways, including transforming growth factor- β (TGF-β) signaling. These findings suggest that plasma EVs from CRS patients induce harmful responses in renal cells by regulating key pathways, highlighting their role in both type 1 and type 2 CRS.

## COMMENTARY

**Figure 1:**
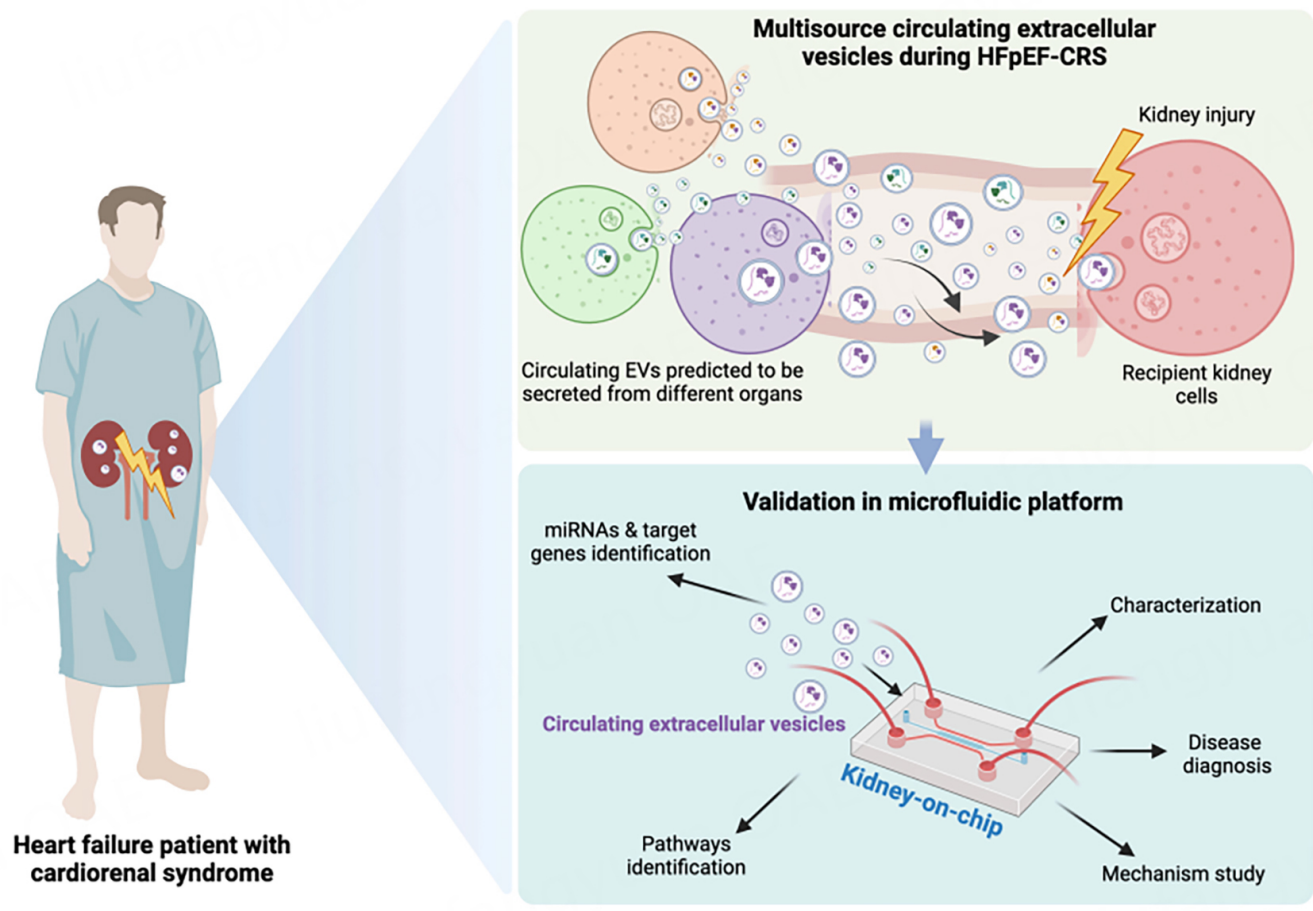
A graphical representation of Renal Injury via Circulating EVs in CRS-HFpEF: A Kidney-on-Chip Analysis (created with BioRender.com). EVs: Extracellular vesicles; CRS: cardiorenal syndrome; HFpEF: heart failure with preserved ejection fraction.

Cardiorenal syndrome (CRS) is a complex condition in which heart and kidney dysfunction are closely linked, increasing the overall morbidity and mortality of these patients. Notably, the presence of both heart failure (HF) with preserved ejection fraction (HFpEF) and chronic kidney disease (CKD) have a poor prognosis due to synergistic effects. Thus, understanding mechanisms associated with CRS is crucial for developing targeted therapies to improve outcomes and reduce healthcare burden. Circulating extracellular vesicles (EVs), small membrane-bound particles carrying a diverse array of molecular cargo, including proteins, lipids, and nucleic acids, play a pivotal role in facilitating systemic EV-mediated communication. Through their molecular payload, EVs can modulate recipient cell behavior, influencing various physiological and pathological processes. Despite their emerging importance in trans-organ communication, the specific role of circulating EVs in CRS remains unknown. In a recent study by Chatterjee *et al*., a “Kidney on Chip”^[1]^ (Emulate. Inc) device was used to investigate the role of EVs in kidney damage associated with HFpEF, shedding light on EV-based mechanisms of renal injury^[2]^. This innovative approach explores the kidney-circulatory system interface, revealing how EVs contribute to renal injury in HFpEF [Figure 1].

The pathophysiology of CRS is complex and not fully understood due to several reasons. Multiple mechanisms such as the renin-angiotensin system, oxidative stress, inflammation, and sympathetic nervous system have been identified as associated factors^[3-5]^, but the precise mechanism remains elusive due to the unavailability of reproducible rodent models. Moreover, there are limitations to using nonhuman primate models due to ethical concerns, high costs, and the risk of zoonotic infections hindering research^[6]^. The development of kidney organoids is obstructed by intricate renal functions and requirements for precise vascularization, drainage integration, and *in vivo*-like microenvironment recapitulation^[3]^. To overcome this, Das group used a human kidney-on-chip model to study trans-organ communication in renal injury mediated by HFpEF patient-derived circulating EVs, offering a translatable approach to understand this complex relationship. Chatterjee *et al*. demonstrated that EVs derived from patients with CRS induce significantly greater renal cell injury compared with EVs from HFpEF patients without CRS^[2]^. This increased injury was evidenced by elevated levels of injury biomarkers, including interleukin-18 (IL-18), kidney injury molecule-1 (KIM-1), and neutrophil gelatinase-associated lipocalin (NGAL). Furthermore, analysis of kidney-on-chip effluents revealed alterations in cystatin C protein expression, a constitutive biomarker of CKD. Changes in cystatin C levels in effluents may parallel *in vivo* fluctuations in circulating cystatin C, reflecting renal function alterations. Notably, the study also revealed activation of profibrotic pathways, specifically transforming growth factor-β (TGF-β) pathway, leading to epithelial-to-mesenchymal transition (EMT) and fibrogenesis. This exacerbates kidney injury, highlighting the potential role of EV-mediated communication in CRS pathogenesis.

EV-derived miRNAs may induce transcriptional and functional changes in recipient cells, suggesting a key role in renal injury^[3,7,8]^. Therefore, establishing a direct link between EV-mediated miRNA signaling and renal injury has the potential to uncover new therapeutic targets. This work identified 78 differentially expressed miRNAs between control and HFpEF patients. Of these, 15 miRNAs were directly associated with circulating creatinine levels, as indicated by the elastic net analysis. These 15 miRNAs targeted 1,143 genes. Pathway analysis revealed enrichment in 35 Kyoto Encyclopedia of Genes and Genomes (KEGG) biological processes, including AMP-activated protein kinase (AMPK) signaling, cell cycle, TGF-β signaling, and O-glycan biosynthesis. Notably, the TGF-β signaling pathway was significantly altered in HF patients, with 7 miRNAs (miR-192-5p, miR-122-5p, miR-146a-5p, miR-629-3p, miR-483-3p, miR-378c, and miR-21-5p) targeting 27 genes. The investigators validated the expression of TGF-β pathway genes in kidney-on-chip cells treated with EVs from healthy controls and HFpEF patients with and without CRS. Specifically, bone morphogenetic protein 6 (BMP6), Follistatin (FST), and tissue inhibitor of metalloproteinases 3 (TIMP3) (targets of miR-192-5p) were downregulated, while epidermal growth factor receptor (EGFR) and SMAD4 (targets of miR-146a-5p) were upregulated in CRS EV-treated group. Additionally, SMAD7 (target of miR-21-5p) was downregulated in CRS EV-treated group. This study highlights the potential role of EV-mediated miRNA transfer in renal dysfunction in HFpEF, particularly through TGF-β signaling pathway perturbation. Furthermore, *in vitro* functional studies revealed that modulating these miRNAs in renal epithelial cells altered downstream targets, including BMP6, TIMP3, SMAD7, EGFR, and SMAD4, and influenced kidney injury markers interleukin 18 (IL-18), NGAL, KIM-1, and cystatin C (CST3). Notably, a miRNA cocktail comprising miR-192-5p/21-5p inhibitors and miR-146a-5p mimic (to oppose the effects of the miRNAs within the CRS EVs) mitigated CRS-EV-induced renal injury, whereas a reciprocal cocktail (miR-192-5p/21-5p mimics and miR-146a-5p inhibitor) phenocopied CRS-EV effects, underscoring the potential of these miRNAs as therapeutic targets for CRS. Given that these miRNAs are carried within circulating EVs of CRS patients, therapeutic approaches could involve the use of EVs as delivery vehicles for miRNA inhibitors/mimics or the development of small-molecule inhibitors that specifically target these miRNAs or their downstream effectors. Thus, the study also highlights the potential of EVs as biomarkers for CRS.

EVs in circulation may originate from many different organs^[9]^, and based on the deconvolution analysis of RNA content of EVs (with respect to tissue atlases of RNA), methods to understand the origin of EVs can be explored^[10]^. However, given the promiscuous expression of miRNAs, the assessment of EV long RNA transcriptome is a better tool to decipher the tissue origin of EVs in the circulation (mRNAs and lncRNAs have better tissue specificity than miRNAs). The deconvolution analysis from Das group’s study reveals that EVs likely originate from the heart, skeletal muscle, adipose tissue, liver and bone marrow (immune cells), with no significant differences between HFpEF CRS and non-CRS groups, suggesting that the miRNA contents of EVs drive disease pathology, rather than its origin. This indicates that circulating EV miRNA profiling provides a non-invasive approach for monitoring CRS disease progression and therapeutic response during HFpEF, addressing the limitations of traditional biomarkers in this heterogeneous disease^[11]^.

## STRENGTHS, LIMITATIONS AND FUTURE DIRECTIONS

The study by Chatterjee *et al*. is substantially strengthened by leveraging human-derived EVs, cells, and particularly the innovative kidney-on-chip model^[2]^. This approach enables a more accurate representation of the complex interplay between organs and EV-mediated communication in CRS. By utilizing this cutting-edge model, the authors aimed to bridge the gap between traditional *in vitro* studies and *in vivo* complexity, providing valuable insights into the molecular mechanisms underlying CRS. Moreover, currently, no established benchmark or study defines the optimal or excessive levels of EV uptake in cells, particularly *in vivo*. Nevertheless, Chatterjee *et al*.’s study^[2]^ provides valuable insights by calculating EV exposure per cell. Notably, their calculations reveal EV exposure levels (6,000 EVs/cell over 72 h) that fall within ranges previously reported to elicit physiological effects^[12]^. Despite its strengths, the study is not without limitations. One major challenge is the inherent complexity of EV biology. EVs are heterogeneous, encompassing a range of particles, including exosomes, microvesicles, and apoptotic bodies, each with distinct biogenesis pathways and cargo. The use of complementary isolation techniques, while enhancing EV specificity, does not entirely preclude the co-isolation of non-EV entities. Therefore, more refined purification approaches are essential to ensure that observed biological effects are exclusively attributable to EVs, rather than to other plasma components that may be present in the isolated fraction. The study’s reliance on in vitro models is another limitation, as they cannot fully capture the intricate physiological processes occurring in living organisms. Although the Kidney-on-chip model is sophisticated, it lacks the cellular diversity and dynamic interactions present *in vivo*. Furthermore, the study does not investigate EV biodistribution in living systems, which is a critical factor in evaluating their therapeutic potential. Consequently, future studies should aim to validate these findings in animal models and, ultimately, in human clinical trials. The study also raises important questions regarding the generalizability of its findings. While the focus on HFpEF provides valuable insights, it is unclear whether the same mechanisms apply to other forms of HF, such as HF with reduced ejection fraction (HFrEF). Further investigation is needed to determine if the identified miRNA signatures are unique to CRS or represent a more universal response to kidney damage. The functional assays employed in this study are restricted to EV uptake and biomarker expression, without direct measurement of cellular behavior alterations. Future research can address this limitation by incorporating additional assays to evaluate cell migration, proliferation, signaling pathways, and functional process analysis, which will provide a more comprehensive understanding of EV-induced effects. Although Chatterjee *et al*. demonstrate that circulating EV miRNAs in CRS patients exert chronic harmful effects on renal cells via TGF-β pathway regulation, the study does not differentiate between type 1 and type 2 CRS, implying possible shared mechanisms^[2]^. Moreover, the clinical relevance of EVs in CRS is still underexplored. While EVs have been identified as potential biomarkers for CRS, their diagnostic and prognostic value in human patients remains unclear due to a lack of large-scale clinical studies. The specificity and sensitivity of EVs as biomarkers need to be thoroughly validated in diverse patient populations, considering factors such as age, gender, and comorbidities. Additionally, the therapeutic potential of EVs, while promising, is hindered by the lack of targeted delivery methods and concerns regarding the safety and scalability of EV-based therapies. Finally, the exact mechanism of how EVs are taken up by the cells and their molecular cargos reach their final destination [e.g., loading onto RNA-induced silencing complex (RISC) complex for miRNAs] is still unclear. Nonetheless, the use of the miRNA mimics and inhibitors to oppose or replicate the effects of the CRS-EV miRNA cargo argues for the functional transfer of these miRNAs and silencing of their target mRNAs.

## CONCLUSION

Studying EVs in CRS offers promising opportunities for advancing our understanding and treatment of the disease. The framework established by Das group’s study provides a robust foundation for investigating EV-mediated communication in multiorgan diseases. However, to fully leverage EVs, we must develop standardized methods, conduct rigorous clinical trials, and explore innovative therapies to overcome existing challenges and unlock their potential in CRS research and treatment.

## DECLARATIONS

### Copyright

© The Author(s) 2024.
